# Strengthening Health Systems to Support Children with Neurodevelopmental Disabilities in Fiji—A Commentary

**DOI:** 10.3390/ijerph17030972

**Published:** 2020-02-04

**Authors:** Sue Woolfenden, Kate Milner, Kali Tora, Kelera Naulumatua, Reapi Mataika, Fleur Smith, Raghu Lingam, Joseph Kado, Ilisapeci Tuibeqa

**Affiliations:** 1 Population Child Health Group, University of New South Wales, Sydney 2031, Australia; r.lingam@unsw.edu.au; 2 Department of Community Child Health, Sydney Children’s Hospital Network, Sydney 2031, Australia; 3 Neurodevelopment & Disability, Royal Children’s Hospital, Melbourne 3052, Australia; Kate.M.Milner@rch.org.au; 4 Neurodisability & Rehabilitation Research & Melbourne Children’s Global Health, Murdoch Children’s Research Institute, Melbourne 3052, Australia; 5 Department of Paediatrics, Colonial War Memorial Hospital, Suva, Fiji; laxtora@gmail.com (K.T.); kelera.paedsreg@gmail.com (K.N.); rlmataika@gmail.com (R.M.); joseph_kado@yahoo.co.uk (J.K.); beth.vereti@gmail.com (I.T.); 6 Nossal Institute for Global Health, University of Melbourne, Melbourne 3000, Australia; smith.f@unimelb.edu.au; 7 Telethon Kids Institute, Perth 6009, Australia

**Keywords:** low and middle-income country (LMIC), Fiji, developmental paediatrics, neurodevelopmental disability (NDDs), children

## Abstract

Supporting children with neurodevelopmental disabilities (NDDs) is recognized as an increasing priority in Fiji, a middle-income Pacific Island country. Our objective was to describe our approach to developing a model of care and strengthening local leadership in developmental paediatrics in Fiji to ensure high-quality identification, assessment and management of children with NDDs. Paediatric staff at Colonial War Memorial (CWM) Hospital in Suva have worked in partnership with Australian paediatricians to develop the model of care. The platform of continuing medical education during biannual 3 to 4 days of clinic-based teaching with visiting developmental paediatricians from Australia has been used. Since 2010, there have been 15 local and regional paediatric trainees trained. Since 2015, our two local lead paediatric trainees have run a weekly local developmental clinic. In total, 370 children aged 0 to 18 with NDDs have been comprehensively assessed with a detailed history and standardised tools. The model is extending to two divisional hospitals. Research engagement with the team is resulting in the development of a local evidence base. Local, regional and international leadership and collaboration has resulted in increased capacity in the Fijian health system to support children with NDDs.

## 1. Background

Worldwide, over the last 15 years, there have been marked improvements in the number of children surviving infancy and early childhood in low- and middle-income countries (LMICs), due to social and economic changes and advances in the provision of universal health care [1]. Recognizing this the Global Strategy for Women’s Children’s and Adolescents Health (2016–2030), the pre-eminent global health strategy, has moved beyond survival to broader goals of ensuring that every child is enabled to survive and thrive, reaching their developmental potential. Alongside this, the Sustainable Development Goals (SDGs) launched in 2015 not only have a focus on improved health but health equity and optimal early childhood development, with SDG indicator 4.2.1 in particular relating to the percentage of children under 5 who are developmentally on track in health, learning and psychosocial wellbeing [2]. Key to this global strategy is supporting children with neurodevelopmental disabilities (NDDs). NDDs include, among others language delay, intellectual disability, autism and cerebral palsy (CP) [3]. It is estimated that there are 53 million children with NDDs worldwide with 95% living in LMICs [4,5]. However, this is likely to be an underestimate due to poor availability of disability data, lack of inclusion of many children with NDDs in society and the stigma children and their families experience [4,5,6]. Through their life course, people with NDDs are more likely to have higher levels of morbidity and mortality, complete lower levels of education, be unemployed, and be socially isolated [5]. They are also at increased risk of trauma, abuse and neglect. These adverse outcomes for children with NDDs increase the likelihood of a life lived in poverty, a loss of country productivity and increased health and welfare expenditure costs [5].

If children are to move from surviving to thriving then health systems need to be able to support early identification of NDDs, comprehensive diagnostic assessment and access to high-quality early intervention, preferably in the preschool years, in order promote optimal physical health, socio-emotional and learning outcomes [7,8,9,10,11]. The importance of early identification and early intervention, targeted according to need, has been highlighted by many global bodies, including through countries adopting the Nurturing Care Framework, launched at the World Health Assembly in 2018 [9,12]. The World Bank has also published seminal policy guidelines on the importance of a diagnostic work up of children with NDDs in order to inform intervention plans made with children and their families [13].

Translating these global policies and frameworks into action at national and subnational level through health services for children with NDDs is a challenge in Fiji as in other LMICs. Although there is a clear evidence base for supporting LMIC health systems through policy and workforce development to improve neonatal outcomes, and communicable diseases, the evidence for health systems supporting children with NDDs is in its infancy [10]. A particular challenge relates to available human resources within health [14]. The recent Global Survey of Inclusive Early Childhood Development (IECD) and Early Childhood Intervention (ECI) Programs (https://www.ecdan.org/assets/global-survey-of-iecd-and-eci-programs---2019.pdf) found that a lack of properly trained and qualified personnel and lack of mentoring, coaching and reflective supervision were key barriers to program quality and translation for children with NDDs [15]. In LMICs, clinicians work in a context where there are stretched resources and competing priorities [16]. For example, paediatricians in the Pacific may be providing services for children with NDDs but also dealing with a Dengue epidemic. In such a situation the acutely unwell child is prioritised over the diagnostic assessment of a child with NDDs. There may also be a lack of awareness of the evidence on the importance of early identification, diagnostic assessment and early intervention for NDDs [9]. In addition, much of the training and assessment approach for NDDs in high-income countries is neither feasible due to limited time or resources, nor in some cases culturally appropriate. Program and external expertise; pre- and in-service training; and networking and collaboration are key to ensure successful IECD and ECI programs [15].

In 2007, the World Health Organization (WHO) identified six “building blocks” of an effective health system—service delivery, health workforce, information systems, access to medicines and technologies, financing, and leadership and governance [17]. The service delivery building block encompasses quality of care and service models that are responsive to need and enable equitable access, while the health workforce building block refers to actions for the planning, training, distribution and monitoring of health workers to enable effective service delivery. Despite a clear policy direction promoting the development of leadership and health systems for children with NDDs [9,17], systematic and evidence-based reviews indicate that there is very little in the literature regarding the development of models of care for children with NDDs, particularly in the Pacific Region [10,18,19].

In this paper, we present a commentary to describe a ten-year collaborative effort between the Fijian Paediatric Clinical Services Network and developmental paediatric colleagues in Australia to develop a model of care for children with NDDs in Fiji.

## 2. The Setting—Fiji

**Figure 1:**
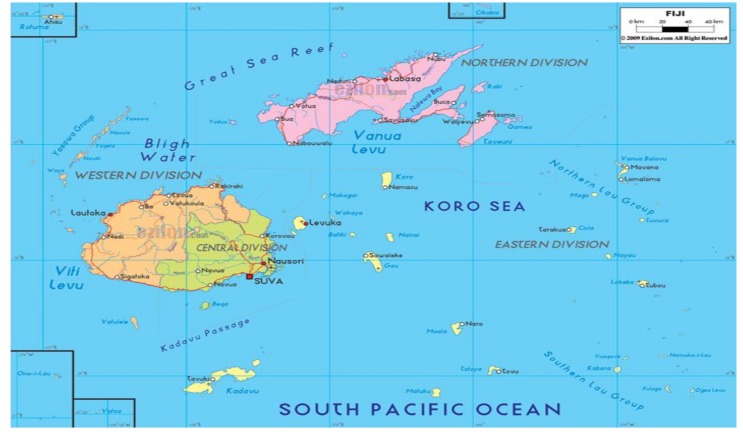
Map of Fiji.

Fiji is a middle-income Pacific Island country with a population of 838,698 people—of which, approximately one-third are 15 years of age or younger [20]. Fiji consists of 322 geographically spread islands (Figure 1), although approximately 75% of the population now live on the main Island of Viti Levu, with the population particularly concentrated in and around the capital Suva [20]. An estimated one-third of the 300 islands in Fiji are inhabited with many remote rural islands and the interior of the larger islands is difficult to access by road or boat. The three predominant languages are English, the official national language, Fijian and Fijian-Hindi. Dominant religions in Fiji are Christianity, Hinduism and Islam [20].

As with many middle-income countries, child health in Fiji is in an epidemiological transition, with an increasing policy and programming emphasis on children with NDDs and inclusive education [21]. Over the past several decades there have been important reductions in the infant and under 5 mortality and the burden of vaccine preventable disease has decreased, although this remains an important cause of childhood mortality and morbidity [22]. The current neonatal mortality rate is 9–10/1000 births [22]. Supporting children with NDDs is an increasing priority for the Fiji Ministries of Health and Medical Services (MoHMs) and the Ministry of Education (MoE). This was exemplified in 2017 by endorsement of the Pasifika Call to Action for Early Child Development by the Government of Fiji in 2017 [23]. On the 15th of June 2017, the United Nations welcomed Fiji’s ratification of the Convention of the Rights of Persons with Disabilities [24].

Colonial War Memorial Hospital (CWMH) is the tertiary paediatric referral hospital for the Fiji Islands and also acts as the sub regional referral paediatric hospital for the South Pacific. It has a paediatric emergency department, a paediatric intensive care unit with 500 admissions/year and a neonatal intensive care unit with 500 admissions/year. There are 9000 births/year. CWMH has 95 inpatient beds but 120 inpatients at times, such as when there are Dengue, Leptospirosis or Typhoid outbreaks after the wet season. CWMH has an outpatient department which sees an estimated 10 000 children a year. CWMH staff also support and link closely with paediatric teams in the two sub-divisional hospitals of Lautoka and Labasa and the team conduct outreach clinics to the Fiji Islands. The paediatric team at CWMH has a total of 20 paediatric medical staff of varying levels of training experience. CMWH is closely linked to Fiji National University which, in 2019, will have 90 medical graduates. There are 10 visiting specialists who come to Fiji to work with local staff including, as we will describe in this paper, developmental paediatrics. Physiotherapy and dietetic services for children exist at CWMH but are more limited or lacking at other sub-divisional hospitals.

Paediatric trainees gain their qualification in paediatrics in Fiji through an initial one-year Diploma of Child Health Program and then a three years Masters in Paediatrics program run by the Fiji National University (FNU). Paediatric trainees in Fiji will often do between one and two years of specialist training in Australia, New Zealand and India in subspecialties. Paediatric staff come from many countries throughout Asia and the Pacific (i.e., Fiji, India, Pakistan, the Philippines, Timor Leste, Tuvalu, Vanuatu, Kiribati, Cook Islands, Nauru, Solomon Islands, Tonga, and Samoa). This means that collaborative partnerships in Fiji have regional implications.

In summary, there are high levels of coverage of acute health care services in Fiji. It is in this context that the partnership with two developmental paediatricians from Australia (SW, KM) began. In 2010, when the partnership began, it was estimated that approximately one child a day was presenting to the outpatient department with suspected NDDs. There was a lack of training and confidence amongst local paediatric staff, a lack of assessment guidelines and a lack of clear referral pathways to early intervention for children with NDDs.

## 3. The Model of Care

**Figure 2:**
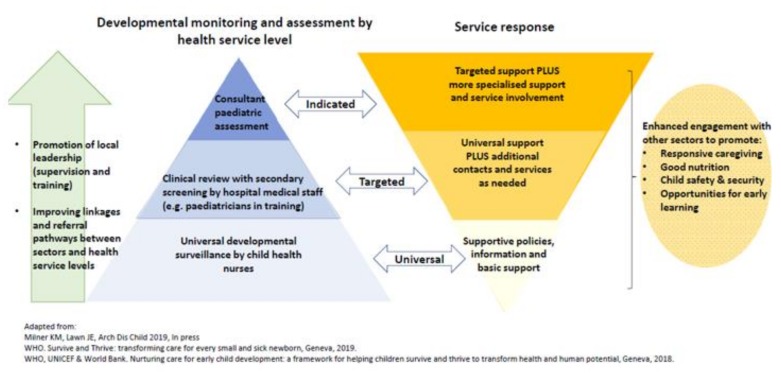
Strengthening health systems in developmental paediatrics in Fiji.

The model of care (Figure 2) developed in this collaboration follows the evidence-based recommended tiered approach to supporting children with NDDs [11,12,25,26]. The developmental paediatric services sits within a broader universal framework of early identification. Our collaboration has supported the establishment of “red flags” for NDDs up to the age of two years on the Fijian Maternal Child Health Card, which is used as part of the universal developmental surveillance system by community nurses. These questions include milestones on mobility, language and self-care. Children identified by this and other services as at risk of NDDs in the Suva-Nausori catchment then come to CWMH and have a clinical review by local paediatric trainees in the outpatient department and at this point may also receive referrals for investigation and early intervention. Other pathways to CWMH include ex-neonatal intensive care/paediatric intensive care patients; children who were self-referred by their parents; children referred by schools; children referred by the local early intervention service. Children with suspected NDDs undergo a diagnostic assessment by our future leaders in developmental paediatrics (local paediatric trainees K.N. and K.T.) with developmental paediatric consultant support (S.W., K.M.) as needed. Children receive a diagnosis and are referred for investigation (including hearing tests, vision tests for all and limited blood tests, neuroimaging as indicated) and early intervention or if school aged, educational support.

The processes in the model have evolved organically since 2010. In 2010, S.W. made her first visit and subsequently has been a visiting developmental paediatrician twice a year staying for 3 to 4 days seeing between 13 and 15 children always with a local Fijian paediatric trainee. The clinic has had support of visiting specialists including speech pathologists, occupational therapists, a paediatric neurologist and a paediatric geneticist. To ensure continuity of care and sustainability of the service, each child has an allocated Fijian paediatric consultant. During the clinics, the local paediatric trainees are taught how to undertake a developmental history and examination using the “see one, do one, teach one method” and a standardised history and examination template from SW’s workplace in Sydney. SW initially used the Griffiths Mental Developmental Scale (GMDS) [27] non-verbal scale as a diagnostic assessment tool. However, in the interests of time and sustainability, this was replaced with a tool that could be used quickly by local paediatric trainees, the Ages and Stages Questionnaire (ASQ) [28]. The ASQ was chosen as it is the developmental screening tool now taught in the undergraduate medical curriculum in Fiji and is available in the outpatient department. The Childhood Autism Rating Scale [29] and Diagnostic and Statistical Manual of Mental Disorders (DSM) V [3] are used to support diagnostic work up of children with suspected Autism Spectrum Disorder. To date, 15 paediatric trainees have been trained using this method. They now assess the children independently and present them to the visiting developmental paediatrician as long cases which then meet their training requirements for the Diploma of Child Health and Masters of Paediatrics. Now all of these paediatric trainees, some who are now consultants, are expected to be able to competently assess and manage a child with suspected NDDs.

**Figure 3:**
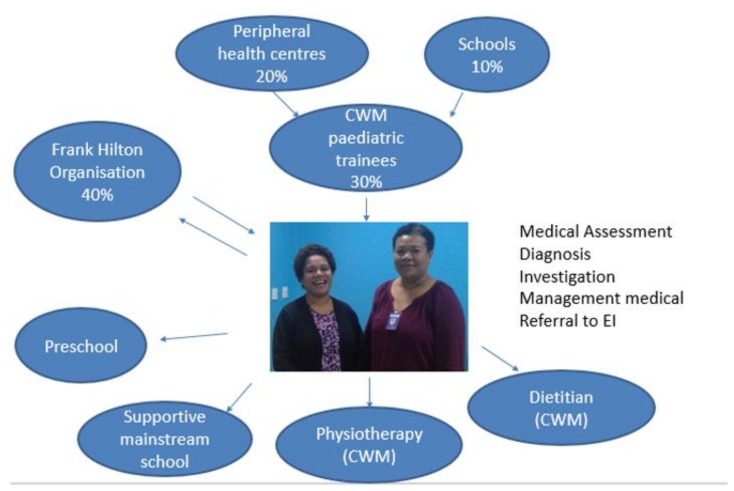
Intake and Referral Pathways to developmental paediatric service*CWM = Colonial War Memorial.

Since 2015, two trainees, K.N. and K.T., have become the nominated leads in developmental paediatrics at CWMH and now have a regular weekly clinic where they see children with suspected NDDs referred from other trainees, community health workers, schools and the local early intervention service. K.N. and K.T. support the development of local trainees and therefore contribute to the development of a sustainable workforce for developmental paediatrics in Fiji and the region. The process is outlined in Figure 3.

**Figure 4:**
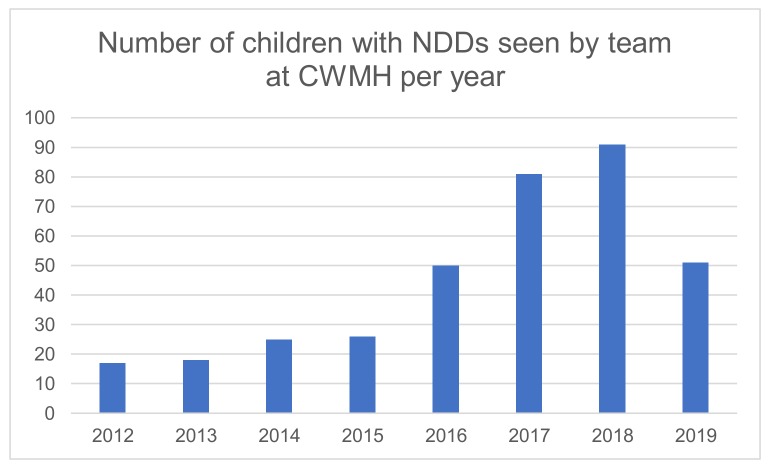
Total number of children with Neurodevelopmental Disabilities (NDDs) seen by the team.

Since 2012, a total of 370 children with NDDs have been assessed at CWMH with a marked increase since the local paediatric trainee clinic began (data missing 2010/2011) as outlined in Figure 4. Children seen have had a wide range of conditions including; language delay, sensorineural deafness, blindness, genetic syndromes, CP, global developmental delay, learning difficulties, intellectual disability, congenital hypothyroidism, and increasingly autism spectrum disorder. The team is arranging ethics clearance to undertake a detailed audit of the clinic and describe the patients seen in more detail.

As an example of the type of clinical scenarios addressed, a deidentified case study of a child seen in our model of care is outlined in following paragraphs.

X, a 4-year-old male was referred to our local paediatric developmental clinic at CWMH via the Children’s Outpatient Department following parental concerns of unusual behavior and delayed speech with poor social-personal skills.

In this clinic, he was seen by a local senior paediatric trainee, where a diagnosis of Autism Spectrum Disorder with severe language delay was made. The diagnosis was made possible through systematic and comprehensive history taking, physical examination with the assistance of existing assessment tools, for example Childhood Autism Rating Scale (CARS) form and Ages and Stages Questionnaires (ASQ).

X subsequently underwent blood investigations (full blood count, thyroid function tests), audiometry screening, vision testing with a referral to his local special school for early intervention. Family counselling was undertaken with regards to the diagnosis, prognosis and importance of supportive care. X was also seen with the visiting developmental paediatrician from Australia to reaffirm the diagnosis, as well as to provide X’s parents an opportunity to clarify any doubts on Autism Spectrum Disorder.

### 3.1. Early Intervention in Fiji

A key opportunity and challenge is the current early intervention offered in Fiji. The two key referral services for the developmental paediatric team in Fiji are the physiotherapy department at CWMH and the Frank Hilton Organisation (FHO). The physiotherapy service provides therapy to children with NDDs and a playgroup for children with CP, however staff training in paediatrics generally and specifically in needs for children with NDDs vary substantially. Additionally, aides and assistive devices for children with NDDs are limited as are orthopaedic services. Currently, there are no paediatric speech pathologists, psychologists or occupational therapists employed by the MoHMs nor training courses for these in the three universities in Fiji.

The Frank Hilton Organisation (FHO) is a non-government organisation increasingly supported by the MoE and has been operating to provide services for children with NDDs in Fiji for over 50 years [30]. The FHO includes an Early Intervention Centre (EIC) in Suva, in close proximity to CWMH. All children attending the FHO have individual family service plans and over the past year or so the EIC has been beginning to run play-based therapy groups for young children with NDDs. Leadership development for long-term local staff at the EIC is an identified priority for the FHO

### 3.2. Embedding Research in Service Ddevelopment

A key component of our work in Fiji is to build a local evidence base and research partnerships in NDDs. Collaboration between Fiji MoHMs and medical services staff and our group of Australian paediatricians, led by KM recently provided the first long-term neurodevelopmental outcome data for Neonatal Intensive Care Unit (NICU) in Fiji [31]. This research examined the outcomes of children discharged from the CWMH NICU and a matched cohort of non NICU babies to examine the prevalence of moderate to severe NDDs. Being a high-risk neonate, gestational age, birth weight, asphyxia, meningitis and/or respiratory distress were significantly associated with risk of NDDs [31]. Prevalence of NDDs was high among this predominantly term high-risk neonatal cohort compared with controls [31]. These results have informed efforts to strengthen the quality of care and our model and have now led to a pilot trial to improve the health and wellbeing of children with CP and their families. The “Toso Vata” (Moving Together) pilot parent support and early intervention program is a local an adaptation of “Getting to Know CP” [32], an evidence-based facilitated participatory learning support program for parents. In March 2019, the team met with a group of multisectoral stakeholders to plan its trial which will be completed in December 2019.

In 2020, K.N., one of our local leaders, is planning to conduct a mixed methods evaluation with the team’s support of the model of care for children with NDDs. This will include qualitative interviews of caregivers about their experiences attending the clinics and their recommendations for future redesign. We are also planning to interview the paediatric trainees regarding their training experience in this health system. The quantitative component will include a detailed audit of the demographic, clinical and service use characteristics of the children with NDDs who have attended the developmental paediatric clinics over the last 10 years. We will use the evidence from this evaluation to develop approaches to address identified barriers to health care access and to support further training and ongoing professional development of local allied health staff and ancillary services (e.g., mental health, orthopaedics). This evaluation will also be an important Fijian led and much needed contribution to the evidence base for models of care for children with NDDs in LMICs.

## 4. Challenges for the Future

A recent systematic review highlighted that the way forward to provide an integrated evidence-based model of care for children with NDDs in LMICs requires early identification linked to early intervention, a whole family approach and “collaborative child health and development partnerships” [11]. This article has outlined our approach to laying the groundwork for this in Fiji. To further strengthen this model of care, we need to consider the feasibility of our approach in terms of human resource capacity, diagnostic tools that are appropriate to the LMIC context, training needs and reach of early identification and early intervention.

In terms of human resources, adequate time to assess and manage children with NDDs (30 to 45 min) versus daily and urgent clinical responsibilities is an ongoing tension. There is also a lack of capacity for a multidisciplinary clinic. This is exacerbated by fact that the countries which have with higher rates of children with NDDs (LMICs) have the lowest number of paediatricians and allied health staff required to assess and treat children with NDDs [16].

Finding feasible and accurate screening and diagnostic assessment tools in the LMIC context is a challenge, as recently outlined in two systematic reviews [25,33]. Our clinics originally used the GMDS [27] to assess a child’s developmental age, which was done by the visiting developmental paediatrician. Due to time and training constraints it was decided by the team that instead of a diagnostic assessment tool such as the GMDS, the locally available developmental screening tool, the ASQ [28], would be used. This is done in conjunction with the Childhood Autism Rating Scale [29] and DSM V [3], as is clinically appropriate. These tools have been validated in LMICs but not for the Pacific region and the ASQ is a screening tool only thus limiting the assessment [33].

There is also a need to strengthen pathways for early identification and intervention with regards to management of NDDs [9]. This entails enhancing community nurse training in child development monitoring with data to monitor referral patterns and gaps; ongoing integration between health system levels and across sectors including education and social services; increased incorporation of data related to child development into the health information system [11]. We need to support approaches to early intervention that are feasible, sustainable and effective in context, have an ongoing supply of assistive devices and provide broader support for parents [9]. There are challenges with accessing early intervention outside of Suva and variation in the support and care in mainstream and special schools. Both the FHO and the MoHMs are strengthening linkages and referral pathways between health and education. CWMH through KT KN and IT are working closely with referrers, physiotherapy and the FHO to develop referral pathways, training and case conferencing.

As with all child health services, there are also challenges in reaching and engaging those children who are likely to most need the services who live in squatter settlements, regional areas and remote villages. We have started to address this by extending the training out to the two divisional hospitals in Labasa and Lautoka.

## 5. What Has been the Benefit?

For children with NDDs and their families in Fiji, there has been access to high-quality developmental paediatric services. For the Fijian team members, there has been on-the-ground clinical teaching, mentoring and career development support and a meaningful collaboration in further service, policy and research. There has been increasing regional capacity by sensitizing and training regional trainees which has been shown in other middle-income countries to increase knowledge, perceived competence and skills related to assessing children with NDDs [34]. For the Australian team members, there has been significant experience within resource-limited settings, promoting clinical skills, innovation, resourcefulness and efficiencies working with local and regional team to provide services for children with NDDs. This reciprocal benefit from global ways of working has been consistently shown to be a benefit in the literature [35,36].

## 6. Conclusions

If we are to truly meet the global challenge of moving children from surviving to thriving, we must strengthen health systems for children with NDDs by collaboratively developing service delivery models and increasing workforce capacity. Key to this collaboration are developmental paediatric services that are appropriate to context, based on shared knowledge and reciprocal learning between experts and local staff, that are high quality and sustainable. We have shared our experience from Fiji of trust, mutual respect and in-country training, making our model resilient, adaptable and sustainable to meet the needs of children with NDDs and their families.
